# Supplemental fish oil decreases urinary excretion of a marker of bone resorption in healthy adults

**DOI:** 10.1186/1550-2783-8-S1-P14

**Published:** 2011-11-07

**Authors:** Eric E Noreen, Josef Brandauer, Megan H MacNabb

**Affiliations:** 1 Department of Health Sciences. Gettysburg College, Gettysburg PA, USA

## Background

Incorporation of fish oil (FO) into the diet of rodents has been shown to result in positive changes in bone health. Currently it is poorly understood if FO has the same effects on bone health in humans. The purpose of this study was to determine the effects of supplemental FO on levels of urinary N-terminal cross-linked telopeptide (NTx), which is a marker of bone breakdown, and how this is related to the morning levels of salivary cortisol and urinary excretion of interleukin 6 (IL-6).

## Methods

A total of twenty-eight females and twelve males(35 ± 13yrs; 69.1 ± 14.1kg; 29.4 ± 9.2% body fat; mean ± SD) participated in this study. All testing was conducted in the morning following an overnight fast. Baseline measurements of salivary cortisol were collected via passive drool and baseline measurements of urinary NTxand IL-6 were collected from the second void of the day and corrected for creatinine excretion. After baseline testing, subjects were assigned randomly in a double blind manner to one of two groups: 4 g/d of Safflower Oil (SO) or 4 g/d of FO supplying 1,600 mg/d eicosapentaenoic acid (EPA) and 800 mg/d docosahexaenoic acid (DHA). All tests were repeated following 6wk of treatment. A treatment by time, repeated measures ANOVA was used to evaluate differences between groups over time, and a standard Pearson’s r was used to evaluate correlations. Additionally, within group pre-post differences were evaluated using a repeated measures t-test. For all analysis, the alpha level was set at p<0.05.

## Results

Compared to the SO group, there was a significant decrease in urinary creatinine corrected NTx excretion following FO treatment (SO = 17.5 ± 42.9 BCE/mM; FO = -11.3 ± 27.7 BCE/mM; p=0.02). There was also a tendency for urinary creatinine corrected IL-6 excretion (SO = -0.08 ± 1.18pg/mg; FO = -1.8 ± 3.8 pg/mg; p=0.08), and salivary cortisol (SO = 0.029±0.283 µg/dL; FO = -0.069 ± 0.144 µg/dL; p=0.13) to decrease following FO treatment.When analyzed independently, however, there was a significant pre-post reduction for salivary cortisol in the FO group (p=0.04), with no change in the SO group (p=0.68), as well as a significant reduction pre-post for urinary IL-6 in the FO group (p=0.05), with no change in the SO group (p=0.78). However, the change in urinary NTx concentrationwas not related to the change insalivary cortisol concentration(r=-0.017, p=0.9), or the change in urinary IL-6 concentration (r=-0.323, p=0.26).

## Conclusions

Six weeks of supplementation with FO in adults significantly decreased urinary NTx excretion, but this change was not related to changes in cortisol or IL-6.

## Funding

Gettysburg College Research and Professional Development Grant and Genuine Health Corporation.

